# Evidence against the Detectability of a Hippocampal Place Code Using Functional Magnetic Resonance Imaging

**DOI:** 10.1523/ENEURO.0177-18.2018

**Published:** 2018-09-07

**Authors:** Christopher R. Nolan, Joyce M.G. Vromen, Allen Cheung, Oliver Baumann

**Affiliations:** 1 Queensland Brain Institute, The University of Queensland, Brisbane, Queensland, Australia; 2 Interdisciplinary Centre for the Artificial Mind, Bond University, Gold Coast 4226, Queensland, Australia

**Keywords:** fMRI, hippocampus, MVPA, navigation, place cells

## Abstract

Individual hippocampal neurons selectively increase their firing rates in specific spatial locations. As a population, these neurons provide a decodable representation of space that is robust against changes to sensory- and path-related cues. This neural code is sparse and distributed, theoretically rendering it undetectable with population recording methods such as functional magnetic resonance imaging (fMRI). Existing studies nonetheless report decoding spatial codes in the human hippocampus using such techniques. Here we present results from a virtual navigation experiment in humans in which we eliminated visual- and path-related confounds and statistical limitations present in existing studies, ensuring that any positive decoding results would represent a voxel-place code. Consistent with theoretical arguments derived from electrophysiological data and contrary to existing fMRI studies, our results show that although participants were fully oriented during the navigation task, there was no statistical evidence for a place code.

## Significance Statement

More than four decades of research have demonstrated that hippocampal place cells in the mammalian brain play a central role in representing the spatial environment. Their encoding of location is both sparse and anatomically distributed, theoretically rendering it undetectable with population recording methods such as functional magnetic resonance imaging (fMRI). Here we present results showing that if visual confounds and statistical shortcomings are carefully eliminated, there is no evidence for the detectability of a human hippocampal place code using fMRI. Moreover, we discuss in detail how these confounds, among others, are manifest in existing studies and are themselves enough to produce false-positive results. Our findings have important implications for research on mental representations of space.

## Introduction

Acquisition of declarative memories is dependent on the hippocampus. Place cells—hippocampal principal cells that exhibit spatial tuning during navigation—provide a clear behavioral correlate with which to interrogate the neuronal dynamics of this region (O’Keefe and Dostrovsky, 1971). Initially discovered in rodents, the existence of place cells has since been found in other species, including humans (Ekstrom et al., 2003). The activity across populations of such cells, as measured with single-cell recordings, can be decoded to provide an accurate estimate of an animal’s current position (Brown et al., 1998), and the activity appears to reflect a cognitive map, resilient against changes in any internal or external cue. However, the sparse firing and random distribution of spatial tuning among the place cell population suggest that any such place code should be impenetrable to current mass imaging technology such as fMRI.

We are aware of four studies that claim to provide evidence for a voxel place code (Hassabis et al., 2009; Kim et al., 2017; Rodriguez, 2010; Sulpizio et al., 2014). Each experiment involved distinguishing between fMRI scans taken at two or more locations in a virtual arena. All four experiments failed to remove potential visual confounds, either in the form of salient visual landmarks during navigation to a target (Hassabis et al., 2009; Kim et al., 2017; Rodriguez, 2010) or at the target (Rodriguez, 2010; Sulpizio et al., 2014) or as visual panoramas unique to each target location (Kim et al., 2017). We later discuss how these potential confounds, among others, are manifest in each experiment (see Discussion), but note here that any legitimate voxel codes in these experiments could be purely sensory-driven rather than place codes.

Beyond experimental design issues, detecting a voxel place code necessitates distinguishing between complex multivariate voxel patterns. Each of the existing four studies uses multivariate pattern analysis (MVPA) techniques to classify voxel patterns as characteristic of particular virtual locations. We identified several statistical and analytic issues in these existing studies, including contamination of cross-validation training stimuli with test stimuli and falsely assuming activity independence between neighboring voxels, which marred the interpretation of any potential evidence (see Methods and Results). Furthermore, statistical inferences based on MVPA results cannot necessarily rely on classical assumptions, such as inferring group prevalence using standard second-level *t* tests (Allefeld et al., 2016). Such information-like measures also violate assumptions of Gaussian or other symmetric null distributions (Stelzer et al., 2013; Brodersen et al., 2013).

**Figure 1.**
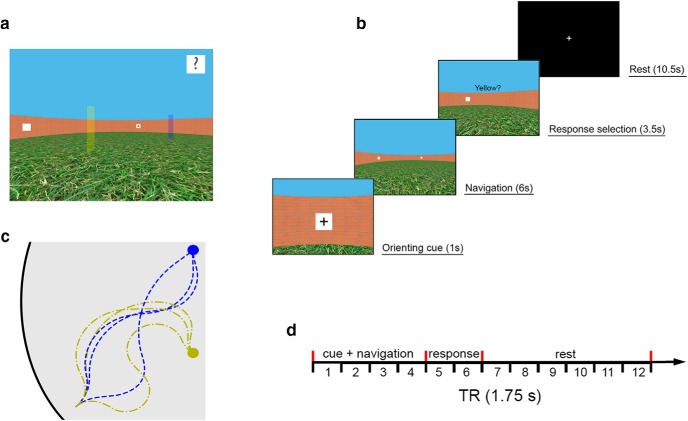
Schematics of the virtual environment and task. ***a***, First-person view of the environment during the training stage (beacons marking target locations are not visible in the main experiment). ***b***, Sequence of events in a typical experimental trial. ***c***, Schematics of the path structures used in the experiment. Participants were led to the target location via in total 24 (three paths from each landmark to each beacon) different curvilinear paths of equal length. ***d***, Experimental time course of each trial relative to the image acquisition sequence (1.75 s per volume).

These concerns motivated us to revisit the question of whether a voxel place code is truly detectable with human fMRI. We had a group of healthy participants perform a virtual navigation task while undergoing high-resolution 3T fMRI. The environment was a circular arena containing two unmarked target locations (see Fig. 1*a*). On each trial, participants were initially shown an orienting landmark and then had to track their position while being passively moved along a curvilinear path to one of the two target locations. During navigation, the participants had to rely solely on their mental representation of the environment and track their position using visual self-motion cues. After arriving at one of the target locations, we probed the participants’ positional knowledge. We then used linear and nonlinear multivoxel classification methods to test whether we could distinguish hippocampal fMRI signals corresponding to periods at which participants were present at each of the two target locations.

## Materials and Methods

### Participants

Twenty-one healthy, adult volunteers gave their written informed consent to participate in the study, which was approved by the Human Research Ethics Committee of the University of Queensland. The first two participants were used only for pilot testing, to optimize acquisition parameters. One participant was omitted from the data analysis because the behavioral performance was below our required accuracy criterion (see Behavioral performance). The remaining 18 participants (9 females) ranged in age from 18 to 29 years (mean, 21 years), and all were right-handed. Classical sample-size estimation techniques are not applicable to the classification analyses in the present study; however, we deemed our sample size sufficient given that three of the four existing studies reported a positive place code effect with fewer subjects (Hassabis et al., 2009; Rodriguez, 2010; Sulpizio et al., 2014).

### Stimuli and procedure

The virtual environment was a circular arena surrounded by a brick wall, with a grass-textured floor and featureless blue sky. The arena wall was 3.0 m high, and its diameter was 30.4 m, relative to a 1.7 m observer. Along the wall, four landmarks (white 1.0 × 1.0 m squares with black symbols: +, %, ?, and #) were located equidistantly (45°, 135°, 225°, and 315°). The two beacons (yellow and blue, see Fig. 1*a*) were 3 m tall and 0.5 m in diameter, located at 0° and 180°, and 5 m from the center of the arena (i.e., 10 m apart from each other).

The task required participants to track their location, while being passively moved (4.2 m/s linear speed) in the absence of orienting landmarks through the environment, therefore relying only on a combination of visual self-motion cues and their mental representation of the landmarks’ locations (see Fig. 1*b* and Video 1). At the beginning of each trial, participants closely faced one of the four peripheral landmarks on the arena wall for 1 s (i.e., the cue card period). Subsequently, all four landmarks were made invisible (i.e., replaced by white placeholders), and participants were turned around and moved for 6 s along a curvilinear path to one of the two unmarked target locations. Participants were led to the target location via 24 different curvilinear paths of equal length (see Fig. 1*c*), so that participants could not infer the target location simply based on the initial landmark cue and the length of the path. After arriving at the target location, participants were prompted to indicate their location within 3.5 s, via a yes/no button response to the question “Yellow?” or “Blue?”, chosen at random. This procedure ensured that the button response was orthogonal to the target location. The response period was followed by a 10.5 s rest period, in which only a white fixation cross on a black screen was shown (see Fig. 1*b*). There were in total 120 trials (60 per target location) split up into five imaging runs, lasting ∼8.5 min each.

**Video 1.** Single exemplary trial of the navigation task.

Although passive movement may degrade place codes, several of the existing studies demonstrating voxel place codes used passive or even static paradigms. An active paradigm could itself introduce several confounds related to the nature and duration of the path that would connect a starting location to the target location. By using a passive paradigm, we were able to ensure the path duration was identical, and thus independent of the distance between starting location and target location, and that the hippocampal code would be spatial (i.e., reflecting the position relative to the configuration of the arena) and not just reflect a combination of start location and path duration or other nonspatial cues. It is important to note that the training of our task included two active navigation phases as well, which should aid the development of the spatial mnemonic representation.

We used the Blender open-source three-dimensional content creation suite (The Blender Foundation) to create the virtual maze and administer the task. Stimuli were presented on a PC connected to a liquid crystal display projector (1280 × 980-pixel resolution) that back-projected stimuli onto a screen located at the head end of the scanner bed. Participants lay on their back within the bore of the magnet and viewed the stimuli via a mirror that reflected the images displayed on the screen. The distance to the screen was 90 cm (12 cm from eyes to mirror), and the visible part of the screen encompassed ∼22.0°×16.4° of visual angle (35.5 × 26 cm).

Before conducting fMRI imaging, participants were assessed and trained using a three-stage procedure to ensure an adequate level of task performance, which depends on familiarity with the arena layout. These behavioral training sessions were scheduled 1 to 2 days before the fMRI scanning session. In the first training stage, participants were allowed to freely navigate the virtual environment for 3 min, using a joystick held in their right hand. During this stage, all four wall landmarks and the two beacons that marked the target locations (yellow and blue) were visible. In the second stage of the training, only the two beacons and one of the peripheral landmarks were visible at a time, and the participants’ task was to navigate to the location of one of the other three landmarks, indicated by a small cue (an image of the landmark) at the top of the computer screen. Each participant completed at least 24 trials of this task. The third stage of the training procedure was almost identical to the actual task described earlier, except the yellow and blue beacons marking the two target locations were visible during the first six trials, feedback was provided for 1.5 s after each button press (i.e., “correct”/“incorrect”), and the interval between trials was just 2 s. Each participant completed at least 24 trials of this task. When participants achieved a performance level of >90% correct in the last stage of the training, they were admitted to the fMRI session. At the beginning of the scanning session, during the acquisition of the structural images, participants performed another iteration of the training tasks to refamiliarize them with the environment.

### MRI acquisition

Brain images were acquired on a 3T MR scanner (Trio; Siemens) fitted with a 32-channel head coil. For the functional data, 25 axial slices (voxel size 1.5 × 1.5 × 1.5 mm, 10% distance factor) were acquired using a gradient echo echoplanar T2*-sensitive sequence [repetition time, 1.75 s; echo time, 30.2 ms; flip angle, 73°; acceleration factor (GRAPPA), 2; matrix, 128 × 128; field of view, 190 × 190 mm]. In each of five runs, 294 volumes were acquired for each participant; the first four images were discarded to allow for T1 equilibration. We also acquired a T1-weighted structural MPRAGE scan. To minimize head movement, all participants were stabilized with tightly packed foam padding surrounding the head.

## Data analysis

### Preprocessing

Image preprocessing was conducted using SPM12 (Wellcome Department of Imaging Neuroscience, University College London). Functional data volumes were slice-time corrected and realigned to the first volume. A T2*-weighted mean image of the unsmoothed images was coregistered with the corresponding anatomic T1-weighted image from the same individual. The individual T1 image was used to derive the transformation parameters for the stereotaxic space using the SPM12 template (Montreal Neurologic Institute template), which was then applied to the individual coregistered EPI images.

Two alternative approaches of detrending were used to assess their potential differential effect on decoding performance. (1) To make use of global information about unwanted signals, images were detrended using a voxel-level linear model of the global signal [LMGS; Macey et al. (2004)] to remove high-frequency as well as low-frequency noise components due to scanner drift, respiration, or other possible background signals. (2) To remove spatiotemporally confined signal drift and artifacts, runwise polynomial detrending was performed on region of interest (ROI) data (see below). By default, second-order polynomial detrending was used (SPM, Wellcome Department of Imaging Neuroscience, University College London, London, UK).

Based on existing evidence that in humans the right hippocampus should be the most likely region to produce a place code (Burgess et al., 2002), we used the AAL atlas (Tzourio-Mazoyer et al., 2002) and WFU pickatlas tool (Maldjian et al., 2003) to generate a right hippocampal (RH) ROI mask. For additional control analyses, we also generated ROI masks for the left hippocampus (LH), left parahippocampal gyrus (LPH), and right parahippocampal gyrus (RPH). The masks were separately applied to the 4D time series using Matlab 2015b (Mathworks).

### Multivariate pattern classification

We performed an ROI-based multivariate analysis (Haynes, 2015) designed to test whether fMRI activation patterns in the human hippocampus carry information about the participants’ position in the virtual environment. The fMRI blood oxygen–level dependent (BOLD) signal has an inherent delay relative to stimulus onset of ∼2 s until it increases above baseline, and ∼5 s to peak response (Huettel et al., 2014). To account for this delay, we selected for the analysis the volumes corresponding to the period of 3.5–5.25 s after participants arrived at the target location (i.e., fMRI TR #7 of our 12-TR trial structure; see Fig. 1*d*). The volume selection approach is analogous to that employed by Hassabis et al. (2009) and Rodriguez (2010).

The goal of our multivariate analysis was to test whether we could classify the virtual location of the participant using the selected volumes. The classification was performed using a linear support vector machine (Haynes, 2015), denoted here as LSVM, implemented in Matlab 2015b. Two data sets were constructed, one with correct labels (location 1 or location 2), and one with randomly shuffled labels. Each data set was then randomly partitioned into 10 subgroups (or folds), split evenly between its class labels (stratification). The classifier was trained on 9 folds (training data), and its performance cross-validated on the remaining fold (withheld test data), once for each of the 10 possible combinations of train and test folds. We repeated this procedure 1000 times for each participant (i.e., 1000 random 10-fold stratified cross validations), which allowed us to estimate the distribution of classification accuracy with (true class labels) and without (shuffled class labels) class information, as well as the distribution of classification accuracy associated with randomly partitioning the data, referred to here as partition noise. Estimating a distribution for partition noise is an additional step from standard application of SVM to MVPA, where typically a single partition of the correct label data are used. A major goal of MVPA is to determine if novel multivoxel patterns can be used to predict their true class labels, and there is no way to know a priori how any particular choice of trial assignment among folds affects such predictive capability. Our 1000 random partitions of the data using true class information allows us to characterize this partition noise distribution.

### Positive control and additional verification analyses

As a direct comparison using the same data and preprocessing steps, we replicated the ROI-based SVM analysis to classify two distinct phases within each trial, which we expected to be different at the voxel level (i.e., a positive control). Given that the right hippocampus is known to show task-related activity during spatial navigation tasks (Baumann et al., 2010, 2012; Baumann and Mattingley, 2013), we hypothesized that the hippocampus should express differential fMRI activity patterns during the navigation period of our task compared to the rest period. Taking the delay in the BOLD response into account, we chose fMRI image #4 (navigation) and #12 (rest) of our 12-image trial structure for this comparison (see Fig. 1*d*).

In addition, to eliminate the possibility that negative results could be due to our choice of preprocessing methods, classifier, brain region, or fMRI images (i.e., time to signal peak) we conducted several additional analyses to verify the null results. First, to exclude that a particular choice of signal detrending was suboptimal, we performed the same analysis using both LGMS and 2nd-order polynomial detrending (see Preprocessing). Second, to exclude the possibility that image smoothing may have impaired the discriminability of the fMRI signal, we repeated the analysis using unsmoothed images (Kamitani and Sawahata, 2010). Third, we explored whether there was any decodable signal in the left hippocampus (LH ROI). Fourth, to test whether decoding of location information could be improved by averaging fMRI signals over a longer period (i.e., several images), we conducted analyses averaging two (i.e., images #7 and #8), as well as three consecutive fMRI images (i.e., images #7–#9). In total, this yielded 24 classification analyses. Finally, to investigate whether there could be voxel place codes that are nonlinearly separable, we repeated the same analyses using a radial basis function (Gaussian) SVM (Song et al., 2011), denoted here as RSVM.

### Multivariate searchlight analysis

In addition to the ROI-based classification approach, we also employed so-called searchlight decoding (Kriegeskorte et al., 2006). In this approach, a classifier is applied to a small, typically spherical, cluster of voxels (i.e., the so-called searchlight). The searchlight is then moved to adjacent locations and the classification repeated. This approach has the advantage that the dimensionality of the feature set is reduced, i.e., the multivariate pattern consists of fewer voxels, making the analysis more sensitive to information contained in small local volumes. We followed the searchlight and detrending methods of Hassabis et al. (2009), using spherical searchlights of 3-voxel radius (comprising a maximum of 121 voxels), on run-wise linearly detrended data. LSVM was applied, using 100 random 10-fold stratified cross-validations for each searchlight, both with and without class label information. Each label shuffle was identical among all searchlights to be compatible with subsequent population inferencing and correction for multiple comparisons (Allefeld et al., 2016).

We further included left and right parahippocampal regions in the searchlight analysis to compute differences in proportions of searchlights exceeding a classification accuracy threshold following Hassabis et al. (2009). This analysis quantifies the difference between the proportion of searchlights in the hippocampal and parahippocampal regions which exceeded the 95th percentile classification threshold computed from shuffled location labels. To determine if the difference in proportions was greater than expected by chance, Hassabis et al. (2009) estimated the standard error of the difference-of-proportions using a standard result, implicitly assuming statistical independence between searchlight accuracies [but see Evaluation of analysis used in Hassabis et al. (2009) for further details on the problems of this assumption]. Due to the computing load, this analysis was implemented in Python v3.5 on a 300-node cluster.

### Population inference using a permutation-based approach

For population inference, we followed the nonparametric, permutation-based approach of Allefeld et al. (2016), who provided strong arguments that the random-effects analysis implemented by the commonly used *t* test fails to provide population inference in the case of classification accuracy or other information-like measures, because the true value of such measures can never be below chance level, rendering it effectively a fixed-effects analysis. The reason is that the mean classification accuracy will be above chance as soon as there is an above-chance effect in only one person in the sample. As a result, *t* tests on accuracies will with high probability yield “significant” results although only a small minority of participants in the population shows above-chance classification.

A further advantage of the approach of Allefeld et al. (2016) is the ability to estimate the population prevalence when the prevalence null hypothesis is rejected. This enables direct quantification of the generalizability of a positive finding in the population.

Briefly, first-level permutations (within-participant) were classification accuracies where class labels are randomly shuffled, together with one classification accuracy with correct labels. Second-level permutations (between-participant) were random combinations of first-level permutations across participants, with one of the second-level permutations consisting of accuracies from all correct labels (to avoid *p*-values of zero). The minimum statistic was used across subjects for each comparison (e.g., searchlight or ROI), and for each second-level permutation. For each second-level permutation, the maximum statistic across comparisons was computed to correct for multiple comparisons (Allefeld et al., 2016; Nichols and Holmes, 2001). Since the maximum statistic does not depend on the amount or nature of statistical dependence between comparisons, it is applicable to classification accuracies of overlapping regions such as searchlights (Allefeld et al., 2016; Nichols and Holmes, 2001). By the same reasoning, it is also applicable to multiple comparisons across different analyses of the same ROI, such as SVM classification following different preprocessing methods. Here, we computed the maximum statistic across all ROIs and preprocessing methods (Extended analysis of negative results), and also the maximum statistic across searchlights in each ROI (Multivariate searchlight analysis).

### Stochastic binomial model for shuffled labels

We developed a stochastic binomial model of classification accuracy based on the null hypothesis and cross-validation analysis parameters. Each test volume was assumed to be classified stochastically with classification success governed only by the null hypothesis probability *p*_0_. For *k*-fold cross-validation (*k*-fold CV), there are *n_f_* = *N_T_*/*k* binary choices for each of *k* folds, averaged to give the accuracy of a single partition set (stratified, nonoverlapping hold-out sets). Assuming the training data are entirely devoid of information, then performance on test data must be at chance, i.e.,

(1) $$X∼B\left(x;1,p_{0}\right).$$

The sample probability of a successful prediction per fold is the number of successful predictions averaged over each fold, i.e.,

(2) $$S=\bar{X}=\frac{1}{n_{f}}\sum_{i=1}^{i=n_{f}}X_{i}.$$

Then the variance of the prediction success per trial is

(3) $$V\left(S\right)=V\left(\frac{1}{n_{f}}\overset{i=n_{f}}{\underset{i=1}{\sum }}X_{i}\right)=\frac{1}{n_{f}{}^{2}}\left(\overset{i=n_{f}}{\underset{i=1}{\sum }}V\left(X_{i}\right)\right)=\frac{1}{n_{f}{}^{2}}n_{f}{\;p}_{0}\left(1-p_{0}\right)=\frac{p_{0}\left(1-p_{0}\right)}{n_{f}},$$

assuming statistical independence between scores within a fold. For truly random partitions and large *N_T_*, this seems a good approximation since volumes in close temporal proximity are rare. Thus if the training data are not informative, then the test data are all essentially independent.

The SVM’s *k*-fold CV accuracy from each random partition is the prediction success averaged over all *k* folds. It is tempting to estimate the variance of the average prediction success as

(4) $$V_{null}\left(\overset{¯}{S}\right)=V_{null}\left(\frac{1}{k}\overset{i=k}{\underset{i=1}{\sum }}S_{i}\right)=\frac{1}{k^{2}}\overset{i=k}{\underset{i=1}{\sum }}V\left(S_{i}\right)=\frac{1}{k}V\left(S\right)=\frac{p_{0}\left(1-p_{0}\right)}{n_{f}k}=\frac{p_{0}\left(1-p_{0}\right)}{N_{T}},$$

by assuming that folds are statistically independent. The problem is that although folds are predicted based on uninformative training data, uninformative is not the same as independent. This is because two training sets overlap by (*N_T_* – 2*n_f_*)/(*N_T_* – *n_f_*), since the data points are drawn from the same set.

The more general form of Equation 4 accounts for covariance terms, i.e.,

(5) $$\begin{array}{l} V_{null}\left(\overset{¯}{S}\right)=V_{null}\left(\frac{1}{k}\overset{i=k}{\underset{i=1}{\sum }}S_{i}\right)=\frac{1}{k^{2}}V\left(\overset{i=k}{\underset{i=1}{\sum }}S_{i}\right)\\ =\frac{1}{k^{2}}\left[\overset{i=k}{\underset{i=1}{\sum }}V\left(S_{i}\right)+\underset{i\neq j}{\sum }Cov\left(S_{i},S_{j}\right)\right]\\ =\frac{1}{k^{2}}\left[\frac{kp_{0}\left(1-p_{0}\right)}{n_{f}}+\underset{i\neq j}{\sum }\rho V\left(S\right)\right]\\ =\frac{1}{k^{2}}\left[\frac{kp_{0}\left(1-p_{0}\right)}{n_{f}}+\rho \left(k-1\right)k\frac{p_{0}\left(1-p_{0}\right)}{n_{f}}\right]\\ =\frac{p_{0}\left(1-p_{0}\right)}{N_{T}}\left[1+\rho \left(k-1\right)\right] \end{array},$$

where the correlation coefficient is

(6) $$\rho =\frac{Cov\left(S_{i},S_{j}\right)}{V\left(S\right)},$$

remembering that *V* (*S_i_*) = *V* (*S_j_*) = *V* (*S*). Thus the variance of the null distribution can be written as a function of the null hypothesis probability *p*_0_ and the CV parameters, i.e.,

(7) $$V_{null}\left(\bar{S}\right)=V_{null}\left(p_{0},\theta \right),$$

where the CV parameter *θ* = (*N_T_*, *k*). At present, the correlation coefficient is found empirically assuming each voxel’s signal is independent, normally distributed random noise. Using synthetic noise data instead of fMRI data guarantees there is no classifiable signal in keeping with the null hypothesis, and also enables predictions to be made when designing new experiments. We generated 10^5^ noise data sets, *n_vox_* = 3053 (for RH), *N_T_* = 120, *k* = 10. Using LSVM, *ρ* = 0.0741.

For computational efficiency, we used a Gaussian approximation of the binomial model:

(8) $$f_{null}\left(\bar{S}|p_{0},\theta \right)=\frac{1}{\sqrt{2\pi V_{null}\left(p_{0},\theta \right)}}\exp\left[\frac{-{\left(\bar{S}-p_{0}\right)}^{2}}{2V_{null}\left(p_{0},\theta \right)}\right].$$

### Stochastic binomial model for true labels

To model the partition noise of individuals, we cannot model the classification of individual volumes as Bernoulli trials. This is because the partitioning regime ensures that every volume is used once and only once as test data in each random partition set. Since the labels remain unchanged, there is in fact no randomness in terms of the test data, i.e.,

(9) $$\bar{S}=\frac{1}{k}\sum_{i=1}^{i=k}S_{i}=\frac{1}{N_{T}}\sum_{i=1}^{i=N_{T}}X_{i}.$$

No matter how the data are partitioned, the pairing of *X_i_* and its label remains unchanged. Therefore $\bar{S}$ is constant and

(10) $$V\left(\bar{S}\right)=0.$$

The problem here is that although the test data are identical over each complete partition set, the training data vary. That is, for *X_i_* in two partition sets, the corresponding training data differ. This difference creates variability in the classification outcome. For shuffled labels, this variability was irrelevant, since classification outcomes were already assumed to be maximally independent. To account for the training set variability using true labels, we can reframe the problem as one where the test data are the reference, and we model how the training data vary with random partitions. Now the random partitions have substantial overlap so that only a small fraction are truly independent between partition sets. For a given test data point *X_i_*, we can estimate the effective number of independent samples per fold, denoted as $n_{f}\prime$. Following Equation 3,

(11) $$V\left(S\prime \right)=\frac{1}{n_{f}{}^{2}}n_{f}\prime p_{1}\left(1-p_{1}\right),$$

where *p*_1_ denotes the mean probability of success for that data set (volumes and labels combination). Using Equation 11 but otherwise following the same logic as the derivation of Equation 5, the variance of the distribution due to partition noise is estimated by

(12) $$\begin{array}{l} V_{part}\left(\overset{¯}{S}\right)=V_{part}\left(\frac{1}{k}\overset{i=k}{\underset{i=1}{\sum }}S_{i}\right)=\frac{1}{k^{2}}V\left(\overset{i=k}{\underset{i=1}{\sum }}S_{i}\right)\\ =\frac{1}{k^{2}}\left[\overset{i=k}{\underset{i=1}{\sum }}V\left(S_{i}\right)+\underset{i\neq j}{\sum }Cov\left(S_{i},S_{j}\right)\right]\\ =\frac{1}{k^{2}}\left[kV\left(S\prime \right)+\underset{i\neq j}{\sum }\rho V\left(S\prime \right)\right]\\ =\frac{1}{k^{2}}\left[\left(k+\rho \left(k-1\right)k\right)\right]V\left(S\prime \right)\\ =\frac{p_{1}\left(1-p_{1}\right)}{N_{T}}\left[1+\rho \left(k-1\right)\right]\frac{n_{f}\prime }{n_{f}} \end{array}.$$

Now the factor $n_{f}\prime /n_{f}$ is the fraction of data that is independent. Since the problem is reframed as one of variability in training data, the fraction is equivalently expressed as the fraction of training data that is independent, given a test data point *X_i_*. For large *k* and random partitioning, few of the remaining *n_f_* – 1 points in a fold with shared *X_i_* would be the same across partition sets. As a first-order approximation, assume that all *n_f_* – 1 points are different, so that the fraction of distinct, and hence independent, data points in each training set is

(13) $$\frac{n_{f}\prime }{n_{f}}\approx \frac{n_{f}-1}{N_{T}-n_{f}}.$$

Substituting Equation 13 into Equation 12, we get

(14) $$V_{part}\left(\overset{¯}{S}\right)=V_{part}\left(p_{1},\theta \right)\approx \frac{p_{1}\left(1-p_{1}\right)}{N_{T}}\left[1+\rho \left(k-1\right)\right]\frac{n_{f}-1}{N_{T}-n_{f}},$$

where the CV parameter *θ* = (*N_T_*, *k*). For computational efficiency, we used a Gaussian approximation of the binomial model:

(15) $$f_{part}\left(\bar{S}|p_{1},\theta \right)=\frac{1}{\sqrt{2\pi V_{part}\left(p_{1},\theta \right)}}\exp\left[\left(\frac{-{\left(\bar{S}-p_{1}\right)}^{2}}{2V_{part}\left(p_{1},\theta \right)}\right)\right].$$

### Bayes factor analysis

We defined a Bayes factor contrasting an alternative hypothesis with the null hypothesis:

(16) $$BF_{10}=\frac{\int_{{\;p}_{1}}f_{part}\left(\overset{¯}{S}|p_{1},\theta \right)f_{1}\left(p_{1}\right)dp_{1}}{f_{null}\left(\overset{¯}{S}|p_{0},\theta \right)}=\frac{Pr\left(\overset{¯}{S}|H_{1},\theta \right)}{Pr\left(\overset{¯}{S}|H_{0},\theta \right)},$$

where the commonly used subscript _10_ denoting the alternative hypothesis is in the numerator and the null is in the denominator. Using the model for an individual’s true classification (unshuffled labels), we can compute the likelihood for the null hypothesis and the likelihood for the alternative averaged over a prior distribution *f*_1_. The typical prior distribution used is the most uninformative distribution that still converges for the Bayes factor calculation. For open intervals, that is usually the Cauchy distribution. In our case, classification rates cannot exceed 1, so the least-informative distribution is uniform between 0.5 (null) and 1, i.e.,

(17) $$\begin{array}{l} H_{0}:p_{0}=0.5\\ H_{1}:p_{1}\in \left(0.5,1\right] \end{array}.$$

The uniform prior assumes that perfect classification success is equally likely a priori as just above chance. Although using the least informative prior potentially reduces unintended bias in the analysis, it also runs the risk of raising the threshold for finding evidence for the alternative, thereby seemingly favor the null. To test this possibility, two other prior distributions were also used for the alternative hypothesis, namely, a linear and quadratic distribution both maximal at *p* = 0.5 and decreasing to zero at *p* = 1. These distributions weight any alternative hypothesis *p* near 1 as less likely than the uniform prior.

For 0.5 < *p*_1_ ≤ 1, the three prior probability density functions of *p*_1_ used were

(18) $$f_{1}\left(p_{1}\right)=\left\{\begin{array}{cc} 2 & \text{Uniform}\\ 8\left(1-p_{1}\right) & \text{Linear}\\ 24{\left(1-p_{1}\right)}^{2} & \text{Quadratic} \end{array}\right\}.$$

The density functions of Equation 18 were substituted one at a time into Equation 16 and combined with Equation 8 and Equation 15 to estimate the Bayes factor Equation 16. Note that for computing Bayes factor for location classification, *θ* = (120, 10), and for task classification, *θ* = (240, 10).

Assuming that a priori, the null hypothesis and weighted alternative hypothesis are equally likely, i.e., *Pr*(*H*_1_) = *Pr*(*H*_0_), then the Bayes factor is

(19) $$BF_{10}=\frac{\int_{p_{1}}f_{part}\left(\overset{¯}{S_{n_{f}}}|p_{1},\theta \right)f_{1}\left(p_{1}\right)dp_{1}}{f_{null}\left(\overset{¯}{S_{n_{f}}}|p_{0},\theta \right)}=\frac{Pr\left(H_{1}|\overset{¯}{S_{n_{f}}},\theta \right)Pr\left(H_{1}\right)}{Pr\left(H_{0}|\overset{¯}{S_{n_{f}}},\theta \right)Pr\left(H_{0}\right)}=\frac{Pr\left(H_{1}|\overset{¯}{S_{n_{f}}},\theta \right)}{Pr\left(H_{0}|\overset{¯}{S_{n_{f}}},\theta \right)}=\frac{L\left(H_{1}\right)}{L\left(H_{0}\right)},$$

which is the relative likelihood of the alternative hypothesis to the null hypothesis, given the data and CV parameters. Consequently a large BF means more evidence for *H*_1_, and a small BF means more evidence for *H*_0_, as defined by *f_part_*, *f*_1_, and *f_null_*.

## Results

### Behavioral performance

We set a stringent performance criterion of 80.5% accuracy for at least four out of five runs, to ensure that the participants were consistently oriented during the task. The threshold was calculated using *α* = 0.05 with the conservative Bonferroni correction, assuming independent Bernoulli trials (chance performance, *p*_0_ = 0.5), and using a Gaussian approximation, i.e.,

(20) $$\text{threshold}=p_{0}+\sqrt{2}erf^{-1}\left(1-\frac{\alpha }{n}\right)\sqrt{\frac{p_{0}\left(1-p_{0}\right)}{n}},$$

where *n* was 24 trials per run. This was necessary to minimize the possibility that failure to decode location from fMRI data could be due to poorly oriented participants.

The 18 participants included in the fMRI analysis had an average performance accuracy of 96.4 ± 1.0% (mean ± SEM). Remarkably, perfect performance was achieved in 58% (52/90) of runs pooled across all participants. Furthermore, the accuracies for target location 1 (mean ± SEM, 96.9 ± 0.9%) and target location 2 (96.0 ± 1.1%) were indistinguishable (*p* = 0.34, *w*_12_ = 27, Wilcoxon signed rank test).

### Multivariate ROI analysis

**Figure 2.**
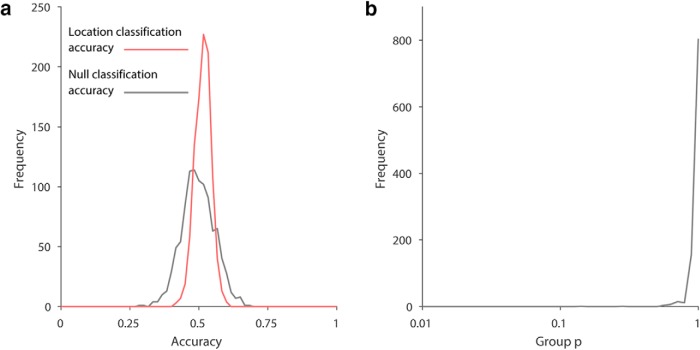
Results from right hippocampus for location classification. ***a***, A typical individual participant’s distribution of classification accuracies (10-fold stratified cross-validation results) for location in the virtual arena, from 1000 random label-shuffles (black) and 1000 random partitions of true labels (red). ***b***, Population inference results for location classification following Allefeld et al. (2016) show no evidence of a place code (18 participants, one *p*-value computed for each of the 1000 random partitions).

**Figure 3.**
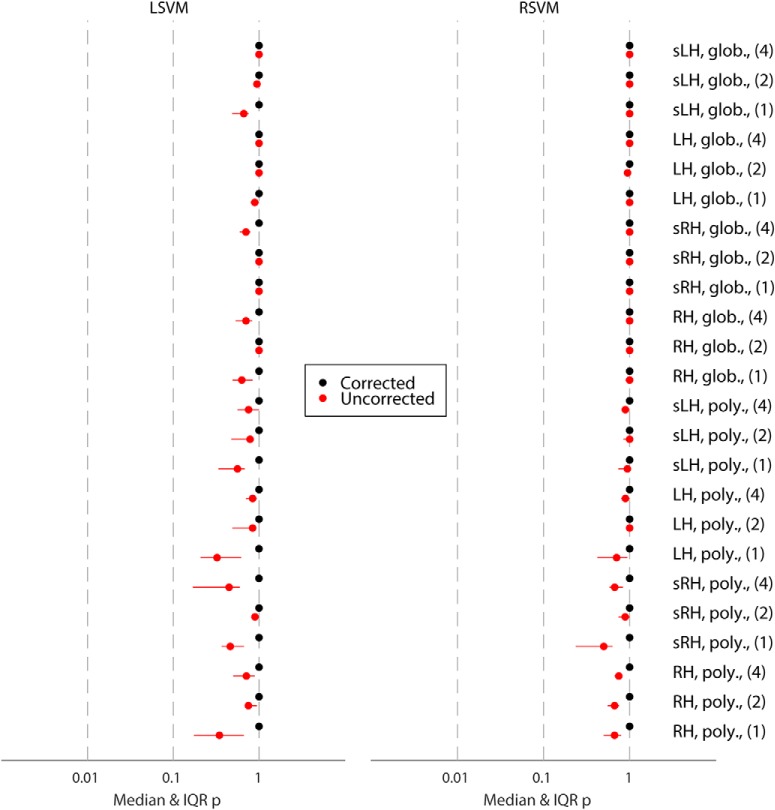
Overview of group significance results for different analysis approaches for the location classification following Allefeld et al. (2016), showing median as well as interquartile range. Glob., linear model of the global signal detrending; H, hippocampus; L, left; R, right; LSVM, linear support vector machine; poly., polynomial detrending (2nd order); RSVM, support vector machine with radial basis function (Gaussian) kernel; s, smoothed (Gaussian kernel, radius = 3 mm). Numerals (i.e., 1, 2, and 4) indicate number of consecutive images used for classification analysis.

Despite behavioral data demonstrating that participants were spatially oriented during the task, the multivoxel classifier could not predict location based on right hippocampal fMRI data. Fig. 2*a* depicts a typical participant’s results for the classification of location, using our default method (i.e., LMGS detrending, 3 mm Gaussian smoothing, LSVM). As expected, the accuracy following random label-shuffles was distributed around the theoretical chance level of 0.5, since the shuffle process removes true location information. If multivoxel patterns were predictive of location in the virtual arena, then accuracies of the unshuffled data sets should be at or beyond the positive extreme of the shuffled distribution. Instead, unshuffled distributions were centered within the shuffled null distribution in all participants, arguing against the presence of location information at the voxel level. Notably, the variability in the unshuffled distribution can be due only to random partitioning itself since the set of unshuffled labels is unique. Thus if only a single partition is used, which is standard practice currently, it is unclear to which part of the partition distribution it might correspond (Fig. 3*a*, red distribution). Therefore, to account for partitioning noise, statistical inferencing using cross-validation methods should be based on a sample of random partitions, or at least incorporate an estimate of partition noise variance. Using the default method, the partition noise variance in our data were 24 ± 2% (mean ± SD, *n* = 18) of the corresponding null distribution variance. For normally distributed independent random variables, if the true null variance is 24% larger than assumed, there would be 7.8% false positives at *p* < 0.05 and 2.1% at *p* < 0.01 (2-tailed false positive % = $100\times \text{erfc}\left[{\text{erf}}^{-1}\left(1-p\right)/\sqrt{1.24}\right]$), potentially inflating false-positive conclusions by 1.5- to 2-fold.

For completeness, we submitted individual classification results from the 18 participants to a group analysis according to Allefeld et al. (2016). The prevalence null hypothesis states that the proportion of participants in the population having an above-chance location classification is zero. Fig. 2*b* shows the group results for our default analysis where the group *p* > 0.1 for all random partitions, consistent with the null hypothesis that there is zero prevalence of location information in the population. Importantly, there was no evidence here that the conclusion may be affected by the instance of random partition of data used for cross-validation.

### Extended analysis of negative results

To investigate whether negative results could be due to our choice of preprocessing method, classifier, brain region, or fMRI images (i.e., time period), we conducted several additional analyses to verify their validity. Fig. 3 shows results for location classification across 24 different analysis approaches, including an alternative preprocessing method (second-order runwise polynomial detrending), varying the number of consecutive images used for analysis, including left hippocampus, and including RSVM in addition to LSVM. Using LSVM, the median corrected group-level *p*-value for the location classification under the prevalence null hypothesis exceeded 0.05 in all cases (Fig. 3, left). In fact, even the lower limit of the 95% confidence interval of the *p*-value (arising from partition noise) exceeded 0.05. The same was true using RSVM (Fig. 3, right). Our results also discount the possibility of a very weak but genuine voxel code that is by some means lost through the correction for multiple comparisons, since the median uncorrected *p*-value was never close to 0.05 (all *p* > 0.3). Therefore, no evidence for a classifiable voxel code for location was found, despite >96% mean behavioral orientation accuracy. Notably, there was no evidence that any particular choice of preprocessing method, classifier, ROI, or timing made a significant improvement to location classification accuracy.

### Multivariate searchlight analysis

One possibility for a negative result may have been the “curse of dimensionality,” because the data dimensionality (e.g., 3053 voxels in right hippocampus) is substantially higher than the number of data points available for classification (e.g., 60 visits to each location per participant). In fact, for both RSVM and LSVM, we found <1 classification error out of 120 when no data were withheld during training (averaged over participants, ROIs, and preprocessing methods), showing that the problem was indeed of generalization to untrained data, rather than the separability of training data per se.

By restricting each classification problem to a small subregion of the ROI, searchlight analysis substantially reduces the data dimensionality and has the potential to partially mitigate the dimensionality problem. Following Hassabis et al. (2009), we applied LSVM to spherical searchlights centered on each voxel in right and left hippocampus and right and left parahippocampal gyrus (see Methods for details). This analysis produced 100 (cross-validation) accuracy values for each voxel of each ROI of each participant, using shuffled labels. Additionally, we produced an equivalent set of results from 100 random partitions of unshuffled data (for each voxel of each ROI of each participant).

Next we looked for evidence of a place code in any individual participants’ results using a nonparametric permutation analysis method (Nichols and Holmes 2001). This approach avoids the need to make a priori assumptions about the data (which is implicit if statistical parametric maps are used). Beginning with the searchlight classification accuracy results, over each ROI, the maximum classification accuracy was found for each shuffled data set and for each random partition of the unshuffled data set. We then found the number of random partitions (out of 100) for which the maximum statistic of the unshuffled searchlight results exceeded the 95% threshold of the shuffled searchlight results. If there is no signal, approximately five partitions should exceed the 95% threshold by chance. Across all ROIs, the mean number of partitions above the 95% threshold did not exceed 5/100 (mean ± SEM/100, RH = 3.2 ± 0.7, LH = 2.5 ± 0.8, RPH = 3.7 ± 0.7, LPH = 4.1 ± 1.1), showing no evidence of above-chance classification for location. We then asked whether it was possible that there could be a weak place signal which for some reason did not reach the arbitrary threshold of 95% of the shuffled data’s maximum statistic. We tested this possibility by counting the number of shuffled maximum statistics that each random partition’s unshuffled maximum statistic exceeded. The presence of a positive bias (>50%) may still suggest a weak but genuine place signal. Instead, no positive bias was found in any ROI (mean ± SEM, RH = 45 ± 3%, LH = 41 ± 3%, RPH = 44 ± 3%, LPH = 44 ± 3%).

In addition to the individual analysis, we also performed a group permutation test following Allefeld et al. (2016). Permutation-based information prevalence inference using the minimum statistic was used to determine if there is statistical evidence for a location code in the population (see Table 1). We started with the same searchlight classification accuracy results as above. In contrast to individual analysis, the minimum statistic was first found for all searchlights across participants, in each ROI. We used 10,000 2nd-level permutations, each of which was a random sample of one shuffled data set from each participant (one permutation was the unshuffled data). The minimum accuracy was found across participants, for each searchlight of each permutation.

**Table 1.** Group permutation test results showing the number of voxels for which *p* < 0.05 in each ROI, averaged across 18 participants

ROI	No. voxels (*p* < 0.05, uncorrected; mean ± SD)	No. voxels (*p* < 0.05, corrected; mean ± SD)	Total no. voxels (common to all participants)
RH	74 ± 14	0.01 ± 0.10	2533
LH	91 ± 17	0.01 ± 0.10	2505
RPH	73 ± 14	0.01 ± 0.10	2157
LPH	59 ± 14	0.02 ± 0.14	1720

For each voxel, the uncorrected *p*-value was the fraction of permutation values of the minimum accuracy that was larger than or equal to the unshuffled data. Hence if the unshuffled accuracy is very high, very few of the permutation values will exceed it (low *p*-value). Since one permutation was the unshuffled data, the minimum *p*-value was 10^–4^. Even without correction for multiple comparisons, we found *p* < 0.05 in fewer than 4% of voxels in each ROI.

To correct for multiple comparisons (multiple searchlights), the maximum statistic (across searchlights) of the minimum accuracy (across participants) was computed. The *p*-value of the spatially extended global null hypothesis was the fraction of permutations in which the maximum statistic was larger than or equal to the unshuffled data. Across all random partitions, on average <1 voxel reached *p* < 0.05 in each ROI (Table 1). Taken together, both uncorrected and corrected group results argue against the presence of location information in the searchlight accuracy values.

There remain a number of possible reasons that a place signal may not have been detected using the ROI-based and searchlight-based multivariate classification methods described. One possibility is that the signal-to-noise ratio is too small to allow signal detection given the size of the training sets used for the classifier, or the number of participants tested in the case of group results. This is unlikely to explain the null finding, since a number of studies have been reported that seemingly showed a voxel-level place signal using even fewer training points per participant, and fewer participants overall (Hassabis et al., 2009; Kim et al., 2017; Rodriguez, 2010; Sulpizio et al., 2014). Another possibility is that the analysis itself may be suboptimal for detecting this type of signal. To test this second possibility, we applied the difference-of-proportions analysis of Hassabis et al. (2009) to our searchlight accuracy values.

First, 10-fold stratified cross-validation results were pooled across all voxels in each ROI over 100 replications where location labels were randomly shuffled. This represents a null distribution of searchlight-based classification accuracy values, devoid of location information. For each ROI, the number of unshuffled voxels whose classification accuracy exceeded the 95th percentile of the pooled distribution was found (Hassabis et al., 2009). The difference in the proportions of suprathreshold voxels was computed between all ROI pairs. According to Hassabis et al. (2009), finding a single proportion from each ROI avoids the problem of multiple comparisons across many searchlights within each ROI. We therefore replicated the analysis of Hassabis et al. (2009) immediately below, but show later that the implicit assumption of independence between searchlights is flawed.

**Figure 4.**
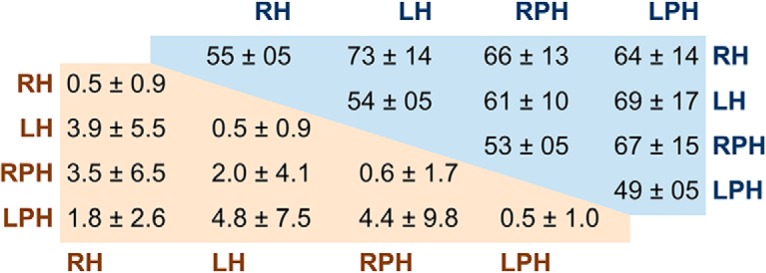
Percentage of ROI contrasts with *p* < 0.05 (top-right) difference-of-proportions method, 10,000 contrast pairs per participant, 18 participants (bottom-left) using shuffled data to estimate standard error of suprathreshold proportions, 10,000 contrast pairs per participant, 18 participants. Note: the two half-matrices are each symmetric around their diagonal; redundant cells have been omitted.

Surprisingly, approximately half of all ROI contrasts resulted in *p* < 0.05 (Fig. 4). This suggests that the proportions of suprathreshold voxels differed between ROIs more than might be expected by chance. If the analysis is valid, this result may well imply that a multivariate voxel pattern exists in some (yet unexplained) location- and ROI-dependent manner. However, by virtue of including 100 random partitions, we could apply the same method to contrast two instances of the same ROI (diagonal cells of top-right section of Fig. 4). Clearly, a valid test should not detect a significant difference between the suprathreshold proportions arising from two random partitions of identical unshuffled data from the same ROI. Yet even for the same ROI, about half of all contrasts had *p* < 0.05. This suggests that the false-positive rate is at least an order of magnitude higher than it ought to be. On more careful inspection of the statistical methods used by Hassabis et al. (2009), it becomes evident that the major reason is an underestimation of the test statistic’s standard error.

### Evaluation of analysis used in Hassabis et al. (2009)

Hassabis et al. (2009) compared the proportions of suprathreshold voxels identified through their standard searchlight analysis, from different ROI pairs. They then employed a commonly used formula (Daniel and Terrell, 1994) to estimate the standard error of the difference between two proportions, namely,

(21) SE^p=p(1−p)(1n1+1n2),

where the pooled proportion *p* is estimated by

(22) $$\hat{p}=\frac{n_{1}p_{1}+n_{2}p_{2}}{n_{1}+n_{2}},$$

where *n*_1_ and *n*_2_ are the numbers of voxels in the two regions being contrasted, and *p*_1_ and *p*_2_ are the proportions of suprathreshold voxels in those regions. Using the estimated standard error from Equation 21, a *Z*-statistic was found which was then used to estimate the probability of a type I error.

Using the estimated standard error from Equation 21 is incorrect here because the implicit assumption that independent Bernoulli-type outcomes contributed to the proportions being compared is violated. The proportion of suprathreshold voxels depends on the number of searchlights whose classification rates exceeded some threshold. However, each searchlight consists of a subpopulation of voxels, with substantial overlap with neighboring searchlights. Therefore, the information in searchlights cannot be considered as independent. Indeed if one searchlight shows high classification accuracy, neighboring searchlights that consist of many of the same voxels are also likely to show similar classification rates. In addition to the overlap of voxels between searchlights, neighboring voxels themselves are known to show correlated activity due to physiology (e.g., shared blood flow) and preprocessing (e.g., low-pass filtering; Poldrack et al., 2011). Empirically, we found a clear positive correlation between the classification accuracies of neighboring voxels in right hippocampus (*r* = 0.72), right parahippocampal gyrus (*r* = 0.74), left hippocampus (*r* = 0.74), and left parahippocampal gyrus (*r* = 0.74). Neighboring voxels were those centered no more than one voxel width away (i.e., maximum of eight neighbors) and within the same ROI mask. Correlations were computed between the mean accuracies of neighboring voxels and the accuracies of the actual voxels themselves.

The assumption of independence between voxels therefore neglects the positive correlation between voxels, which leads to underestimation of the standard error of the difference in suprathreshold proportions. This in turn leads to underestimation of the probability of a type I error. To test if the underestimation of the standard error of the difference-of-proportions was the major reason for the high percentage of ROI contrasts with *p* < 0.05 (Fig. 4), we re-estimated the standard error directly using the shuffled searchlight data. Using the same thresholding method as before, we computed 100 different suprathreshold proportions for each ROI (corresponding to all the shuffled data). Hence, for each ROI contrast, there were 100 difference-of-proportion values from shuffled data, used to estimate the mean and standard error of the null difference-of-proportions for that ROI contrast. For the same ROI pair (e.g., RH versus RH), the standard error was estimated as the RMS of the other ROI pairs involving that ROI (e.g., RH versus LH, RH versus RPH, RH versus LPH). As before, a *Z*-statistic was calculated, and a two-tailed *p*-value estimated using a normal approximation. Using this simple estimate of the standard error of the difference of suprathreshold proportions, the mean percentage of ROI contrasts with *p* < 0.05 dropped to <5% (Fig. 4, bottom left). These results show that by using a more direct estimate of the standard error of difference-of-proportions, the percentage of contrasts with *p* < 0.05 is no more than expected by chance, arguing against an ROI-specific place code.

### Simulating searchlight analysis used in Hassabis et al. (2009) employing independent noise

It is unclear how much of the correlation of searchlight accuracies is a result of searchlight overlaps per se, and how much is a result of other factors such as shared blood flow or low-pass filtering which produces correlations in BOLD signal. It may be that overlaps between neighboring searchlights contribute minimally to the underestimation of the standard error. If so, the problem should not exist if the underlying voxel data are truly independent. To investigate this possibility, we repeated the analysis of Hassabis et al. (2009) on pure noise. We generated 100 independent synthetic data sets by using Gaussian noise of the same mean, standard deviation, and spatial distribution as voxels in our human fMRI ROIs, assuming statistical independence between all voxels. Analysis parameters were the same as for fMRI data. Note that the synthetic data sets were genuinely independent rather than merely using label shuffles as is the case for fMRI data. Since there was no true signal, we systematically excluded one data set at a time to simulate “unshuffled” data (which should not be classifiable). By pooling the voxels from the remaining 99 data sets, we set the 95th percentile threshold for classification accuracy as before. The number of voxels exceeding threshold in each of 100 unshuffled data sets were used along with pooled proportions, and the standard error of pooled proportions, to calculate *Z*-statistics. Using Gaussian approximation, we estimated 2-tailed *p*-values of the *Z*-statistics. For each ROI contrast, all 10,000 possible pairs of data sets were used (100 random partitions from each ROI).

**Figure 5.**
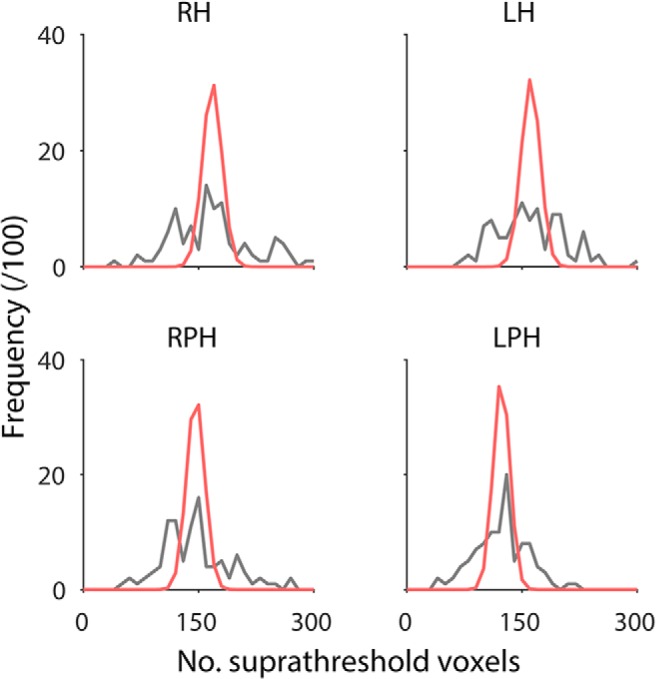
Frequency distribution of suprathreshold voxels in synthetic noise data sets corresponding to each individual ROI (black line, *n* = 100; see text for details). Using the same mean and assuming independent searchlight accuracies, a Gaussian approximation of the expected frequency of suprathreshold voxels (red line) shows substantial underestimation of the spread of suprathreshold voxel counts, causing an inflation of false positives, i.e., either higher or lower classification accuracies than expected by using the faulty null.

If searchlight overlaps per se do not make a significant contribution to the correlation in searchlight accuracies, then there should be ∼5% false positives (by setting *p* < 0.05) in the synthetic data. Instead, using the method of Hassabis et al. (2009), there were >50% false positives in all ROI contrasts, including same-ROI contrasts (Fig. 5 and Table 2), demonstrating that searchlight overlaps alone inflate false-positive rates by an order of magnitude. Therefore, the searchlight method itself introduces enough correlation between otherwise independent voxels to violate the assumption of independence required to use uncorrected estimates of the difference-of-proportions. Taken together, our theoretical and experimental results demonstrate that the implicit assumption of independence in searchlight analyses by using uncorrected estimates of standard error of difference-of-proportions substantially increases false positives, and must be avoided.

**Table 2.** Percentage of ROI contrasts with *p* < 0.05 (pure noise example, difference-of-proportions method, 10,000 contrast pairs)

%	RH	LH	RPH	LPH
RH	63	61	61	59
LH	61	59	59	56
RPH	61	59	59	58
LPH	59	56	58	55

### Analysis of cue card effect

Despite purposefully keeping a range of key visual features constant including overall shape, size, color, and contrast, the cue card at the start of each navigation period was visually distinct and spatially salient. Hence it is conceivable that the cue card itself may have contributed to a voxel-level code. In turn, such a code may have contaminated or even washed out a weak spatial signal from the target location, thereby causing unsuccessful target location classification. If so, perhaps the initial cue card may be classifiable at above-chance level, instead of the target location. This was not the case (hippocampal ROIs; same preprocessing as described previously; volume 7, volumes 7 and 8, and volumes 7–10 of Fig. 1*d*). We further confirmed that visual cue identity could not be classified as a direct response to the visual stimulus onset (L hippocampus, R hippocampus, L lateral occipital cortex, R lateral occipital cortex; same preprocessing as described previously; volume 3, volumes 3 and 4, and volumes 3–6 of Fig. 1*d*). Taken together, these results do not support the possibility that the cue card itself contributed to a contaminating voxel-level code.

### Positive control analyses

**Figure 6.**
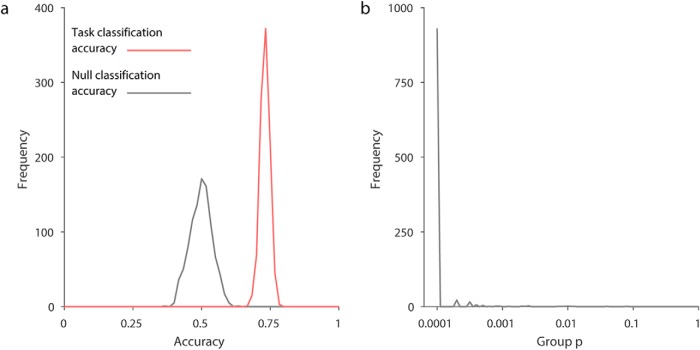
Results from right hippocampus for the control classification. ***a***, A typical individual participant’s distribution of classification accuracies (10-fold stratified cross-validation results) for task type (active versus passive), from 1000 random label-shuffles (black) and 1000 random partitions (red) of true labels. ***b***, Population inference results for control classification following Allefeld et al. (2016; 18 participants, one *p*-value computed for each of the 1000 random partitions).

Since no evidence of a voxel-level place code could be found using a variety of approaches, we investigated the possibility that there was some unforeseen flaw in the image acquisition or analysis protocols. Using the same data, we determined whether two distinct phases in each trial, namely navigation versus rest, could be classified (see Methods). Using our default method (i.e., LSVM, 3-mm smoothing, LMGS detrending), the two phases were clearly separable at a typical individual level (Fig. 6*a*) and at the group level (Fig. 6*b*). These analyses validate our image acquisition and data analysis protocols, and stand in contrast to our unclassifiable location results (Fig. 2).

**Figure 7.**
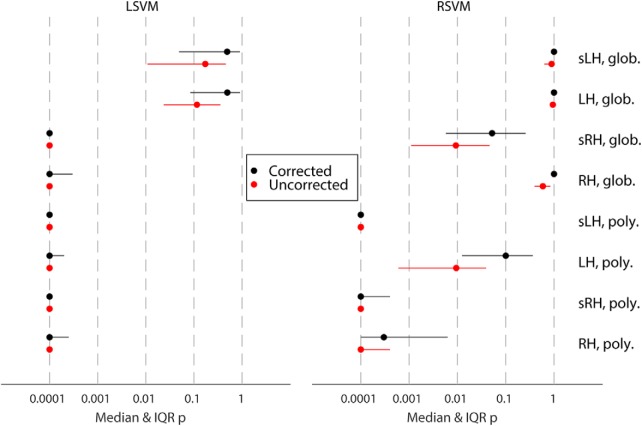
Overview of group significance results for different analysis approaches for the control (i.e., task type) classification following Allefeld et al. (2016), showing median as well as interquartile range. Glob., linear model of the global signal detrending; H, hippocampus; L, left; R, right; LSVM, linear support vector machine; poly., polynomial detrending (2nd order); RSVM, support vector machine with radial basis function (Gaussian) kernel; s, smoothed (Gaussian kernel, radius = 3 mm).

Fig. 7 shows results for the positive control classification across 8 different analysis approaches. The median corrected group-level *p*-value for the prevalence null hypothesis was <0.05 for all navigation-versus-rest-period classifications, across all ROIs, as well as smoothing and detrending methods, using LSVM (see Fig. 7, left). The same was true of RSVM using polynomial detrending (see Fig. 7, right). Note, however, that some 95% confidence intervals for the *p*-values included 0.05, showing that the choice of data partition can significantly affect classification generalization success. Nonetheless, for LSVM even the 97.5th percentile *p*-value was below or close to 0.05 for both left and right hippocampus, using 2nd-order polynomial detrending. Thus at the group level, it is clear that voxel patterns are informative for rest-versus-navigation periods of a task. Furthermore, we can exclude the possibility that only a small proportion of participants had classifiable voxel codes, which biased group results, since for all partitions where the null hypothesis was rejected, we can estimate the 95% confidence interval of the proportion of participants with a classifiable voxel code (Allefeld et al., 2016). For the smoothed right hippocampal data, LSVM resulted in null hypothesis rejection in 999/1000 random partitions. Of those, 0.62–1.00 of all participants are estimated to have a classifiable voxel code for rest versus navigation (95% CI, median of partition shuffles). Taken together, these results suggest that hippocampal voxel patterns can be used to predict rest versus navigation periods at above-chance level, in the majority of participants. Importantly, there is a clear difference between the classification performance for location 1 versus location 2, and rest versus navigation, using the same participants, experimental design, fMRI acquisition parameters, and analysis method.

### Evidence for the null hypothesis

After careful analysis, we did not find any evidence to reject the null hypothesis that there is no voxel place code. However, finding no evidence to reject the null hypothesis is different from finding evidence to directly support it. Therefore, we considered whether the null hypothesis itself can be used to make testable predictions about the fMRI data. We used the default smoothed and globally detrended data from RH to test the predictions.

**Figure 8.**
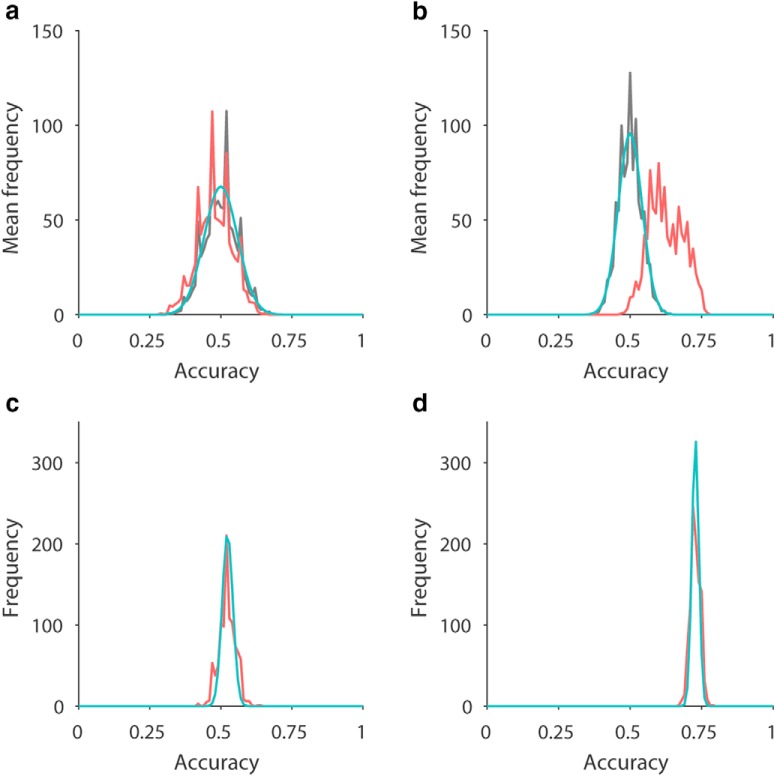
Comparison of noise model and LSVM accuracy distributions from RH. ***a***, The frequency distribution of accuracy results is shown for location classification, averaged across all 18 participants, with shuffled (black) and true (red) location labels. A Gaussian approximation is shown (cyan) using a mean of 0.5 and variance estimated by a stochastic model assuming no label information. ***b***, As per ***a*** but for task classification. ***c***, The frequency distribution of accuracy results is shown for location classification from a typical participant from ***a*** using true location labels (red). A Gaussian approximation is shown (cyan) using the mean of the individual’s sample, and variance estimated by a stochastic model assuming partition noise only. ***d***, As per ***c*** but for task classification.

A straightforward prediction of the null hypothesis is that location labels do not matter and are effectively random when considering a population of participants. Thus for a sufficiently large sample size, the distribution of accuracies arising from true labels should be similar to the distribution due to shuffled labels. This was in fact the case for location classification (Fig. 8*a*, red versus black lines), where even distribution peaks arising from the discrete nature of scores were well matched. This directly supports the null hypothesis, since true location labels were equivalent to shuffled labels and were therefore uninformative. In contrast, if there is a genuine signal, then the two distributions should be distinct, since the pooled distribution using true labels should no longer be equivalent to shuffled labels. This was in fact the case for task classification (Fig. 8*b*, red versus black lines), where the pooled distribution for true labels showed a higher mean and larger variance than for shuffled labels. These differences demonstrate that the true labels were not equivalent to shuffled labels, and therefore task information was present at the voxel level.

Next we asked whether it is possible to derive an approximate form of the pooled distribution for location classification using true labels (Fig. 8*a*, red line), using only the null hypothesis and experimental parameters. If so, this would show that the null hypothesis is a sufficient model to account for the accuracy results, adding further evidence to support the null hypothesis for location classification. To do this, we developed a simple stochastic binomial model of accuracy based on the null hypothesis (see Stochastic binomial model for shuffled labels). Our model was developed assuming statistical independence between data points, which implies no label information. Hence our model should match data if there is no label information. Our stochastic model provided a good match for location classification distribution with either true or shuffled labels (Fig. 8*a*), suggesting that the null hypothesis provides a good quantitative account of location classification data. The stochastic model also predicts that the variance should be inversely related to the number of data points used for classification per participant. For task classification, there were twice as many volumes used for classification (two tasks per navigation sequence), and the pooled distribution for task classification using shuffled labels had a correspondingly smaller variance (Fig. 8*b*).

To more directly contrast the evidence for the null versus alternative hypothesis, we computed Bayes factors for each participant’s accuracy results, using likelihoods estimated using models developed from the hypotheses. Therefore, in addition to the null model above, we needed a model of accuracy scores of individuals with true labels for the alternative hypothesis (that there is genuine information). Following similar arguments as above, we developed a simple stochastic binomial model of accuracy based on fixed labels and random partitions (see Stochastic binomial model for true labels, Fig. 8*c*,*d*). The model depended on the point accuracy score of classification as input and predicted the corresponding accuracy density function. In this way, any prior distribution of accuracies can be used as the alternative hypothesis. To ensure that we did not inadvertently choose an alternative hypothesis that somehow biased outcomes, we tested three different prior distributions of accuracies reflecting varying prior beliefs about true accuracies (see Bayes factor analysis). We employed Bayes factor category thresholds based on Dienes (2014); Jarosz and Wiley (2014); Jeffreys (2000); Ly et al. (2016); Raftery (1995).

There was a consistent pattern showing either no evidence (neutral) or evidence supporting (moderate, strong to extreme) of the null hypothesis for location classification (Table 3, location). In contrast, there was a consistent, but very different pattern showing either no evidence (neutral) or evidence supporting (moderate, strong to extreme) the alternative hypothesis for task classification (Table 3, task). Notably, the same pattern of results persisted across all three prior alternative hypotheses tested.

**Table 3.** Median Bayes factor (from 1000 random partitions), of a total of 18 participants, assumes shuffled labels variance for H_0_

Classification	Prior *p* distribution for H_1_	SH_0_	MH_0_	N	MH_1_	SH_1_
Location	Uniform	8	7	3	0	0
	Linear	5	4	9	0	0
	Quadratic	5	3	10	0	0
Task	Uniform	0	1	6	2	9
	Linear	0	0	6	2	10
	Quadratic	0	0	3	5	10

SH_0_, strong to extreme evidence for H_0_ (*BF* < 1/10); MH_0_, moderate evidence for H_0_ (1/10 ≤ BF < 1/3); N, neutral (1/3 ≤ *BF* ≤ 3); MH_1_, moderate evidence for H_1_ (3 < BF ≤ 10); SH_1_, strong to extreme evidence for H_1_ (10 < *BF*). BF category thresholds are based on Dienes (2014); Jarosz and Wiley (2014); Jeffreys (2000); Ly et al. (2016); Raftery (1995).

Taken together, the convergence of distributional, model, and Bayes factor results directly and consistently support the null hypothesis for location classification and support the alternative hypothesis for task classification. These results complement the nonparametric population inference analyses to argue against evidence for a place code that is detectable using fMRI.

## Discussion

The goal of the present study was to reinvestigate whether human hippocampal place codes are detectable using fMRI. We employed a virtual environment that eliminated any potential visual and path-related confounds during the signal-decoding period to ensure that any positive finding would be indicative of a place code rather than a view code or a conjunctive view-trajectory code. We also employed a variety of signal processing and classification approaches, as well as a positive control analysis to evaluate carefully the possibility of the nonexistence of a spatially driven multivoxel place code.

Our experiment showed that, while participants were fully oriented during the navigation task, there was no statistical evidence for a place code, i.e., we could not distinguish the two target locations using multivoxel-pattern classification algorithms. Additionally, we found robust and consistent evidence to directly support the null hypothesis for location classification data, using Bayes factor analysis and a model of SVM classification results derived from the null hypothesis. These findings support conclusions drawn from electrophysiological rodent data, which suggest that given the sparseness and distributed nature of place codes in the hippocampus, it would be implausible for them to be detectable using fMRI (O’Keefe et al., 1998; Redish and Ekstrom, 2012). A sparse code is one in which relatively little neural activity is used for encoding. All else being equal, sparse codes are more challenging to detect using any measure of local metabolic demands, like BOLD signals, since the signal strength depends on total activity change, which is relatively small. This problem could be alleviated to a degree if place cells across neighboring voxels encode the same location. Unfortunately, place cell codes in rodents have been found to be distributed across the hippocampus, showing no discernible topological relationship with the environment (Redish et al., 2001).

Notably, Eichenbaum et al. (1989) found electrophysiological evidence that place cells within a 1-mm-diameter area show a statistically significant but weak correlation in the location encoded. One possible interpretation is that local ensembles have correlated spatial encoding, leading to the possibility of voxel-level spatial codes. However, numerous scale issues challenge this interpretation. First, each neural ensemble typically encoded the majority of the environment, rendering the spatial specificity of a single recorded ensemble substantially less than a single place cell. Consequently, many pairs of environmental locations would lead to similar ensemble-level activity. Second, typical fMRI voxels have cubic volumes of at least 1.5 mm per edge. Yet each wire in the 10-wire multielectrode used by Eichenbaum et al. (1989) should be able to detect only cells up to 150 µm away (Stratton et al., 2012; Buzsáki, 2004). Hence the total volume recorded would be at most

$$\frac{4\pi }{3}0.15^{3}\times 10=0.14{\text{ mm}}^{3},$$

one to two orders of magnitude less than the smallest voxel typically used in fMRI. If the spatial specificity encoded by such a small neural volume as recorded by Eichenbaum et al. (1989) is already below what electrophysiologists typically set as the spatial selectivity threshold for a place cell (Burgess et al., 2005), the ensemble activity within a full voxel of neural tissue is likely to be well below threshold. Third, because of inherent low-pass filtering of the BOLD signal due to both physiologic and equipment processes (Poldrack et al., 2011), even if there is a weak differential signal in one voxel’s neurons, it is likely to be smeared out across adjacent voxels, meaning that BOLD measurements actually reflect ensemble activity from multiple voxel volumes. Therefore, BOLD signals should be less spatially selective than a single-voxel volume of neurons, which should be less spatially selective than the already subthreshold selectivity of local ensembles. Fourth, even the intrinsic spatial organization of orientation columns in visual cortex, which have a clear cellular organization, has been shown to be identifiable using submillimeter 7T imaging but not with 3T imaging (Yacoub et al., 2008). Taken together, convergent electrophysiological findings of place cells including low ensemble spatial specificity and sparse and distributed coding, along with further evidence against spatial encoding correlations among local neurons in both linear (Redish et al., 2001) and open-field (O’Keefe et al., 1998) environments, argue against the detection of location specific activity using current fMRI technology.

Despite the above arguments, we cannot assume that the ensemble dynamics of a place code in humans are undetectable in fMRI based solely on rodent electrophysiology results, nor can we dismiss the possibility that the organization of spatial information may differ at the resolution of BOLD signals compared to local cell ensemble activity. By the same reasoning, we also cannot make an a priori assumption that finding voxel place codes using fMRI is *fait accompli* simply because rodents show evidence in this regard. Any claim of a voxel place code requires a direct demonstration that it is not tied to specific sensory cues but is rather a fundamental representation of environmental location. If at least part of a voxel code can be unequivocally demonstrated to survive removal of all confounds, then the most consistent and parsimonious conclusion is that a spatial memory of the environment was used. Our experiment was designed specifically to look for such a place code and found evidence only for the null. Our findings are at odds with four prior imaging studies that reportedly have detected multivoxel place codes in the hippocampus (Hassabis et al., 2009; Kim et al., 2017; Rodriguez, 2010; Sulpizio et al., 2014). Since we employed a range of different image preprocessing and analysis approaches, it seems unlikely that our particular choice of analysis strategy could account for the discrepant results. Moreover, our control analysis showed that we were able to detect task-related changes in hippocampal activity, discounting the possibility that differences in image acquisition protocol or potentially image quality could be the reason prohibiting a positive finding.

Considering our results, it is important to carefully identify plausible reasons for the positive fMRI findings of published studies. We identified several limitations in the experimental tasks and analysis strategies of each fMRI study that could explain why each study seemingly detected a multivoxel place code in the hippocampus.

## Statistical concerns

### Invalid assumptions of statistical independence

Hassabis et al. (2009) made the implicit assumption of statistical independence between searchlight accuracies that is violated in fMRI data (see Results). More detailed inspection of the suprathreshold counts from the original experiment (Hassabis, 2009, section 3.6.3) reveals that numerous suprathreshold proportions were in fact <5% despite using a 95th percentile threshold. For example, for their pairwise location comparison for subject 2, the hippocampal suprathreshold count was 118/4032 searchlights (= 2.9%), the parahippocampal gyrus suprathreshold count was 70/3822 searchlights (= 1.8%), and the reported *p*-value was 0.002 for this contrast despite so few searchlights reaching the shuffled data’s threshold. Importantly, all *p*-values reported were replicable using the faulty method outlined earlier. Across 16 contrasts reported, 22/32 suprathreshold proportions were <5%. Therefore, these original results showed no evidence that location classification was possible in either ROI.

### Paired t test on accuracies

Rodriguez (2010) and Sulpizio et al. (2014) relied on a paired *t* test for group analysis of decoding performance. When applied to classification accuracies, such a test will with high probability yield “significant” results although only a small minority of participants in the population shows above-chance classification (see Methods; Allefeld et al., 2016). Hence even a genuine significant result says nothing about the prevalence or generality of the finding.

### Classifier confounds

Rodriguez (2010) included both the encoding and test phases of each trial in the dataset as independent trials. The classifier may have identified the general relatedness of the two phases being part of the same trial, rather than the spatial location per se. Many factors unrelated to location in the virtual arena could have contributed to two consecutive phases of a trial being similar, including simply being close in time.

Similarly, Sulpizio et al. (2014) included several identical images in the training and test sets (i.e., three instances per unique view were used for training the classifier and one for testing it in their leave-one-out cross-validation procedure). This alone could lead to successful overall classification in the absence of a place code.

Finally, Kim et al. (2017) provided few details regarding the path structures used in the navigation task. It is mentioned that only pseudorandom trajectories were used and that 76% of all trials involved the inner eight (out of 64) locations used for the fMRI analysis. It is not clear from the description in which order the locations were visited. The nature of the trajectories could, however, have a significant effect on similarity of the fMRI signals associated with each location, either because of different levels of autocorrelation or different levels of locational awareness that might be confounded with certain path characteristics. In short, without careful quantification of the path structure, it is difficult to exclude the possibility that it might have contributed to the statistical discriminability of the fMRI signal associated with different locations.

### Potential visual confounds

A place code should be demonstrably selective for position in a mnemonic representation of space rather than for position contingent on particular visual cues. If it cannot be ruled out that activity in a region is responsive to visual stimuli, and if an environment does contain spatially specific visual cues, then any spatial response could potentially be due to such cues, and a spatial response cannot be definitively identified as such. Earlier work in monkeys demonstrates that primate hippocampal cells signal locations or objects being looked at, independently of current self-location (e.g., Robertson et al., 1998; Rolls, 1999; Rolls et al., 1997). Human electrophysiology in virtual navigation settings has also shown that individual hippocampal units respond to current view. It is thus imperative that any experiment seeking to identify a place code properly controls for visual confounds. Unfortunately, all four studies that claim to provide evidence for a voxel place code contained potential visual confounds, implying that even a legitimate voxel code in these experiments could be sensory-driven rather than be a place code.

Reliable and unique visual landmarks pose a particular problem. In the most obvious scenario, such a cue might be visible in a period used for classification. The experiment by Sulpizio et al. (2014) required that static visual scenes completely determine location and orientation. Likewise, in the study by Rodriguez (2010), the egocentric view direction of the landmark during navigation varied systematically with the goal location. Furthermore, the virtual environments used by Hassabis et al. (2009) consisted of visually distinct landmarks on or adjacent to all walls, which were not visible during the classification period; however, visual traces or the sluggishness of the BOLD response could contribute to positive classification. The virtual environment outlined by Kim et al. (2017) contained a salient local landmark (a green door). The authors stated that the door was “occasionally” visible, but failed to demonstrate that neither those times nor visual appearance of the door were correlated with impending arrival location. Furthermore, the corresponding analysis compared parallel locations in their rectangular environment, which would undoubtedly provide different panoramas—independent of the allocentric direction—due to different wall distance configurations. In the Kim et al. (2017) study, there was also a connection bias between the locations in the 3D environment employed (i.e., not every location was connected to every location, and connections were not always symmetric) that caused the optic flows to differ depending on which test location was immediately upcoming. Animal studies have shown that the hippocampus is sensitive to visual aspects of linear and rotational motion (O’Mara et al., 1994) and that it receives information from the accessory optic system (Wylie et al., 1999), which is a visual pathway dedicated to the analysis of optic flow. A classifier may be able to detect differences in preceding ground optic flow, which in turn correlated with test location. In summary, in all four of these cases, above-chance decoding could be due to differences in visual information during navigation rather than spatial location.

## Conclusions

All existing studies that assert to have found evidence for a hippocampal place code using fMRI can be challenged based on either statistical or task-related concerns and provide no robust convincing evidence of a multivoxel place code in humans. Further evidence against the detectability of a hippocampal place code using fMRI comes from a published pilot study (*n* = 3) by Op de Beeck et al. (2013), which employed a virtual navigation paradigm with the aim of decoding location information from fMRI activation patterns but also found no statistical evidence for a place code in the hippocampus. They were, however, able to statistically infer spatial location from voxel patterns in the visual cortex, giving further weight to our concerns regarding visual confounds in the aforementioned studies. Moreover, a number of recent studies have shown that patients with hippocampal damage have difficulties in complex visual discrimination tasks, suggesting a role of the hippocampus in visual perception (Hartley et al., 2007; Lee et al., 2005a,b,^2006, 2007^). In contrast, activity of *bona fide* place cells identified in rodents has been shown repeatedly to be view independent and persists even without visual information (Quirk et al., 1990; Rochefort et al., 2011; Save et al., 1998, 2000). Hippocampal place cells of bats have also been shown to persist without visual input (Ulanovsky and Moss, 2007). Place cells identified in the hippocampus of epilepsy patients were also partially view independent because patients’ avatars approached the same virtual location from multiple directions (although the set of visual cues still uniquely defined each position in the virtual environment; Ekstrom et al., 2003). In line with place cell properties common to phylogenetically diverse mammalian species, claiming the existence of a multivoxel place code necessitates exclusion of sensory driven activity differences. A voxel-level neural code is driven by convergent inputs and computations arising from heterogeneous multimodal inputs, and such a code may well correlate with place in an environment. Indeed, locations in real environments are often rich with multimodal sensory cues. However, the richness of spatial information contained in such cues makes it particularly difficult to quantify the extent that different sensory streams contribute to a voxel correlate of place. It is unclear whether a neural representation of place can ever be completely independent of all external sensory correlates of place, despite it being theoretically possible (Cheung, 2014). One avenue to investigate this issue is to determine if putative voxel-level place representations can survive removal of obvious sensory correlates of place.

In summary, we have conducted a detailed assessment of the claim that place codes are detectable using fMRI in the human hippocampus. Our combined experimental and theoretical results provide rigorous and consistent evidence against this claim. Taking our data in combination with the presented theoretical, statistical, and methodological points, we suggest that claims of the existence of a voxel code of location should therefore be treated with appropriate caution. We assert that any future imaging study claiming evidence in favor of a multivoxel place code should rigorously eliminate potential confounds due to visual features, path trajectories, and semantic associations that could lead to decodable differences between spatial locations. In addition, it will be crucial to employ appropriate and robust statistical tools to avoid false positives that are a particular concern for high-dimensional data. We envisage two distinct avenues to further the research beyond our findings here: to systematically explore whether a particular magnitude of visual or semantic information during virtual navigation facilitates successful decoding in hippocampal fMRI; and to test spatial decoding in patients with hippocampal depth electrodes using a task comparable to that presented in the current study. The latter study would identify whether our failure to decode is caused by low spatial resolution of 3T fMRI, or whether because of the virtual nature of the task or species differences, a hippocampal spatial code is not readily accessible in human subjects.

## Synthesis

Reviewing Editor: Bradley Postle, University of Wisconsin Decisions are customarily a result of the Reviewing Editor and the peer reviewers coming together and discussing their recommendations until a consensus is reached. When revisions are invited, a fact-based synthesis statement explaining their decision and outlining what is needed to prepare a revision will be listed below. The following reviewer(s) agreed to reveal their identity: Arne Ekstrom.

Reviewer #1

The manuscript seeks to understand whether voxels in the human hippocampus contain sufficient information to decode a participant's position. 18 participants navigated an environment containing few visual cues (boundary, grass). Participants were trained to find one of two beacons based on four landmarks, but importantly, these were not visible during trials in which they navigated to the locations of one of the two beacons was located. Despite the difficulty of the task, participants navigated with high accuracy. The authors were unable to decode the position of the subject from either the left or right hippocampus using multiple methods, including a search light and ROI approach. In the process, the authors discovered serious flaws with a previous paper that claims to have found place “voxels” in the hippocampus from Hassabis et al. The most major flaw being that the false positive rate in this study was based on an incorrect assumption regarding the independence of search light spheres. Indeed, using this incorrect assumption, that authors found a high level of false positives in randomly simulated noise data. The authors conclude that place selectivity is not decodable from human fMRI data, which follows from the sparsity of place cell firing more generally.

Assessment: This is an important, carefully done, and timely paper. The major advance here, is the inability to decode place selective voxels. This, despite early claims to the contrary, is well backed up here with nicely designed paradigm that eliminates visual confounds (unlike past work, including Hassabis et al). In fact, the flaw of assumption of search light independence is important, and also impacts the fields, in a similar way to papers like the Eklund PNAS paper showing an underestimation of false positive rates in most univariate studies. The methods here are also performed to some of the highest standards in cognitive neuroscience. I have no hesitation about the value of publishing this paper. My only concern currently involves the scholarship in some places. The authors confuse, somewhat, a purely mnemonic code with one that involves some sensory input to retrieve such codes. Even studies in rats that use darkness assume some input, in this case, path integration cues. In fact, when almost all sensory input is removed and a rat is moved through a place cell in a towel, there are no place cells (Foster, Castro et al. 1989). Thus, I suggest fixing some of the statements about mnemonic codes. Some sensory input is necessary for observing place cells in all species, but it is certainly also the case that visual input alone can also produce spurious place voxel responses, given the sluggishness of the BOLD signal. It should also be noted that, contrary to claims from the authors, these issues have been dealt with in VR studies in both monkeys and humans using single cell recordings, by virtue of the more precise time resolution. I detail these and other issues below.

MAJOR

I think the two major issues right now are scholarly. First, the authors need to clarify that, even in rats, SOME sensory input is necessary for seeing place cells, as indicated by the Foster et al. study cited above. The authors should be clear that some partial cues, either visual or vestibular/somatosensory, are necessary to elicit place codes, but in fMRI, this is problematic because of its slow time course. Second, the authors need to correct some misstatements about past work involving place cells recorded in virtual reality in humans and monkeys. They also need to add citations to such work in humans and monkeys. In short, such work in monkeys and humans has de-confounded place from view by using the higher time resolution of single cell recordings and multivariate methods. I strongly suggest reviewing these papers carefully, as I detail below, and correcting misstatements currently in the paper about these issues. I detail these in a point by point manner to make them clearer below.

1) “hippocampal principal cells that exhibit allocentric spatial tuning”

It is incorrect to call place cells allocentric. They also fire on linear tracks, showing egocentric properties. Please see a detailed review paper on this by: Ekstrom, Huffman et al. (2017). The empirical results to focus on are in: Gothard et al. (Gothard, Skaggs et al. 1996)

2) “However, the sparse firing and random distribution of spatial tuning amongst the place cell population suggests that any such place code should be impenetrable to current mass imaging technology such as fMRI.”

This issue has been discussed in some depth in two empirical papers, one in humans and one in rats. The correct citations here are: Redish, Battaglia et al. (2001) and Ekstrom, Suthana et al. (2009). These papers are not currently cited, yet critical to the basis of the arguments here. Thus, I suggest carefully reading and discussing these papers in the current manuscript.

3) “but note here that any legitimate voxel codes in these experiments could be sensory-driven rather than true place codes”

As discussed above, it might be worth nothing that the presence of place cells in the dark does not prove they are mnemonic in rats. Such representations could be supported solely by path integration cues. But I agree that overall, a “true” place code should not depend strongly on sensory input

4) I am confused by Table 1 and the middle label...how is mean+SD deviation of voxels a number .01?

(.01 voxels?)

5) I am confused by Table 2, can this be explained in more detail?

6) It might be worth noting, when discussing why a place code is unlikely to manifest in a BOLD change, that even orientation columns in V1, which have a clear cellular organization, are hard to see in fMRI. They are barely decodable at 7T, but not at 3T (Yacoub, Harel et al. 2008, Dumoulin, Fracasso et al. 2017). This point would seem to bolster the argument that sparse and orthogonally distributed place cells are unlikely to be visible at 3T in humans.

7) “Notably, Eichenbaumet al. (1989) found electrophysiological evidence that place cells within a 1mm diameter area show a statistically significant but weak correlation in the location encoded.”

It is important to note that Redish et al. 2000 (Redish, Battaglia et al. 2001) did not replicate this finding, using a much larger sample set of place cells and studies. I suggest going through this paper carefully here, as well as (Ekstrom, Suthana et al. 2009), reviewing the argument and findings carefully, and discussing them here and elsewhere, as appropriate. They certainly strengthen the authors argument.

8) “and in some cases may be intrinsically interwoven with visual inputs.”

Ekstrom, Kahana et al. (2003) provide a detailed treatment of this issue, showing human place cells are different from view responsive neurons, although there are some conjunctions of the two codes in some cases. For a later detailed treatment of this issue in monkeys, please see: Wirth, Baraduc et al. (2017).

9) “Furthermore, electrophysiological recordings from the human hippocampus suggest that the

majority of active neurons are not spatially-selective, but may instead respond to various types of visual stimuli (Kreiman et al., 2000).

This statement, as written, is incorrect. The Ekstrom et al. 2003 shows a significant number of view-independent place cells in the human hippocampus, around 20%, while the Kreiman et al. paper shows a significantly lower percent of visually responsive cell. Given that there was no navigation or coding of location in the Kreiman et al study, it is unclear why it is even referenced here. It is also not clear that the ”place“ cells in the Kreiman et al. study had anything to do with place but instead scenes. I also suggest later papers showing place cells (not view cells) in humans e.g., (Jacobs, Weidemann et al. 2013, Miller, Neufang et al. 2013). This misstatement above, and the accompanying inferences, should be corrected.

10) ”Setting aside these experimental and statistical concerns, there is a question of whether navigating in VR provide necessary and sufficient stimuli to stabilize place codes at all.“

This statement is contradicted by the three studies mentioned above in humans (Ekstrom, Kahana et al. 2003, Jacobs, Weidemann et al. 2013, Miller, Neufang et al. 2013) , as well as numerous studies done in monkey VR. This statement must be reconsidered given this evidence: (Matsumura, Nishijo et al. 1999, Hori, Tabuchi et al. 2003, Ludvig, Tang et al. 2004), Wirth, Baraduc et al. (2017)

References:

Dumoulin, S. O., A. Fracasso, W. van der Zwaag, J. C. Siero and N. Petridou (2017). ”Ultra-high field MRI: Advancing systems neuroscience towards mesoscopic human brain function.“ Neuroimage.

Ekstrom, A., N. Suthana, D. Millett, I. Fried and S. Bookheimer (2009). ”Correlation Between BOLD fMRI and Theta-Band Local Field Potentials in the Human Hippocampal Area.“ J Neurophysiol 101(5): 2668-2678.

Ekstrom, A. D., D. J. Huffman and M. Starrett (2017). ”Interacting networks of brain regions underlie human spatial navigation: A review and novel synthesis of the literature.“ Journal of Neurophysiology: jn. 00531.02017.

Ekstrom, A. D., M. J. Kahana, J. B. Caplan, T. A. Fields, E. A. Isham, E. L. Newman and I. Fried (2003). ”Cellular networks underlying human spatial navigation.“ Nature 425(6954): 184-188.

Foster, T. C., C. A. Castro and B. L. McNaughton (1989). ”Spatial selectivity of rat hippocampal neurons: dependence on preparedness for movement.“ Science 244(4912): 1580-1582.

Gothard, K. M., W. E. Skaggs, K. M. Moore and B. L. McNaughton (1996). ”Binding of hippocampal CA1 neural activity to multiple reference frames in a landmark-based navigation task.“ J Neurosci 16(2): 823-835.

Hori, E., E. Tabuchi, N. Matsumura, R. Tamura, S. Eifuku, S. Endo, H. Nishijo and T. Ono (2003). ”Representation of place by monkey hippocampal neurons in real and virtual translocation.“ Hippocampus 13(2): 190-196.

Jacobs, J., C. T. Weidemann, J. F. Miller, A. Solway, J. F. Burke, X. X. Wei, N. Suthana, M. R. Sperling, A. D. Sharan, I. Fried and M. J. Kahana (2013). ”Direct recordings of grid-like neuronal activity in human spatial navigation.“ Nat Neurosci 16(9): 1188-1190.

Ludvig, N., H. M. Tang, B. C. Gohil and J. M. Botero (2004). “Detecting location-specific neuronal firing rate increases in the hippocampus of freely-moving monkeys.” Brain Res 1014(1-2): 97-109.

Matsumura, N., H. Nishijo, R. Tamura, S. Eifuku, S. Endo and T. Ono (1999). “Spatial- and task-dependent neuronal responses during real and virtual translocation in the monkey hippocampal formation.” J Neurosci 19(6): 2381-2393.

Miller, J. F., M. Neufang, A. Solway, A. Brandt, M. Trippel, I. Mader, S. Hefft, M. Merkow, S. M. Polyn, J. Jacobs, M. J. Kahana and A. Schulze-Bonhage (2013). “Neural activity in human hippocampal formation reveals the spatial context of retrieved memories.” Science 342(6162): 1111-1114.

Redish, A. D., F. P. Battaglia, M. K. Chawla, A. D. Ekstrom, J. L. Gerrard, P. Lipa, E. S. Rosenzweig, P. F. Worley, J. F. Guzowski, B. L. McNaughton and C. A. Barnes (2001). “Independence of firing correlates of anatomically proximate hippocampal pyramidal cells.” J Neurosci 21(5): RC134.

Wirth, S., P. Baraduc, A. Plante, S. Pinede and J. R. Duhamel (2017). “Gaze-informed, task-situated representation of space in primate hippocampus during virtual navigation.” PLoS Biol 15(2): e2001045.

Yacoub, E., N. Harel and K. Uğurbil (2008). “High-field fMRI unveils orientation columns in humans.” Proceedings of the National Academy of Sciences 105(30): 10607-10612.

Reviewer #2

The authors present a Null Evidence piece that reinvestigates whether human hippocampal place codes are detectible with fMRI.

They tackle a very important issue. I generally agree with their methods, background, and concerns. However, I worry about some aspects of the piece. The authors do not mince words regarding prior imaging studies aimed at detecting place codes, and this may ultimately be quite warranted. I think we should encourage work like this; it does motivate additional care in some aspects. One concern is theoretical, the other is methodological.

Theoretical: The authors lean heavily on place cells, and the finding that they are sparse and not topographically-organized in the rodent hippocampus. I think this is a very good motivation for the present study, but I question whether this emphasis is too strong due to several issues.

-- Has the non-topographically-organized nature of place cells been demonstrated in humans? Can we assume fMRI should fail, given possible species differences? This seems worth considering in the manuscript, especially given that we also know that hippocampal place processing is powerfully influenced by prefrontal control mechanisms and that prefrontal circuitry (and cognition) is markedly different between rodents and humans.

-- In their discussion, the authors briefly acknowledge that the primate hippocampus is very sensitive to visual information. Eichenbaum's work (among others) has underscored that this true in rodents too (see, e.g., https://www.ncbi.nlm.nih.gov/pubmed/24910078). Many “place-like” cells are also sensitive to heading, objects, past experience, motivational states, and exhibit conjunctive response properties. A place cell can even adopt a different type of coding when an animal adopts a different navigational reference frame (Cabral et al., 2014). I wonder if the emphasis on “pure” hippocampal place codes in the field is misguided. To the framing of the current manuscript: does a mnemonic representation of place, if it is indeed place-specific, have to exclude such highly relevant information? It's actively debated whether humans, in real-world settings, regularly rely on path integration mechanisms and how often they ignore landmark information to localize themselves. An interesting discussion point to consider is (https://www.ncbi.nlm.nih.gov/pubmed/25346679). I wonder if a human hippocampal place code that is informed by perceiving a landmark is really problematic to the degree argued by the authors. (given the VR context, the authors may find O'Keefe's recent work - http://www.pnas.org/content/110/1/378 - useful to their points)

-- This prior point is also relevant to the notion that even if “pure” place cells are not topographically-organized in humans, voxel-level asymmetries in response to different locations could certainly arise from population-level convergence of “pure” and “impure” place-relevant and conjunctive-coding place signals. Stripping that information away and finding a null (as shown in the present study) is certainly theoretically important (i.e., can we detect something we'd attribute solely to a pure place cell?), but does not prove that a distributed place code, more broadly defined, is absent from the hippocampus. Is place coding that arises from the convergent, domain-general processing functions of the human hippocampal memory system not place coding? It's worth noting that multi-voxel codes throughout the navigational system appear to leverage visual information to identify distinct landmarks/places and then generalize across marked perceptual differences of those representations (https://www.ncbi.nlm.nih.gov/pubmed/26538658).

This is a lengthy way for me to say I agree with where the authors are coming from and the concerns they raise, but the manuscript may be more constructive if it incorporated more discussion of such considerations and what prior and current (null) outcomes could mean for theories of hippocampal function. Are there research opportunities provided by the outcome of this study?

Methodological: I was generally very impressed with the methodical and thorough approach taken. However, as it is currently presented, I'm not convinced that the paradigm and analyses favor an ability to measure a place code - should it exist.

-- I suspect that cognition plays a key role in spatial signals in fMRI tasks [this may contribute in part to the place code findings in rodents (http://www.pnas.org/content/110/1/378), if we speculate they could potentially “buy in” to the virtual world less than humans (i.e., have less of a sense of presence/that it is equivalent to a physical environment)]. We know from both rodent and human research that passive navigation degrades spatial memory and degrades the place cell signals which are focal point for the study framing. The fact that the authors adopted a passive navigation paradigm is therefore problematic when expecting a null result.

-- The task also does not appear to require much engagement with the goal location at all. There are only two target locations that have well-learned relationships to the orienting landmarks, passive navigation is executed to each goal, and then a simple judgment of A or B at the end of the trial is given. To what degree do the participants really need to track the goal location with any accuracy? It seems wholly sufficient to identify where you are starting, and then determine if you wound up in the North or South hemifield of the environment by attending to your relationship to that landmark and/or start. This could, in fact, force a null result in the present study because if there is a place code it might be largely oriented around monitoring position of the starting landmark, and not the goal.

-- What compels participants to think about their current position in the critical post-arrival analysis period?

-- We know participants were able to indicate whether they wound up in the North or South hemifield with high accuracy. Is there any way to demonstrate that they are thinking about the position with any more spatial precision?

[Editor's note: during the consultation session between the two reviewers and the editor, Reviewer #2 made this comment, which I think concretely articulates one way in which her/his question might be addressed: “I think if they showed an absence of decoding of the goal period based on start landmark/loc, regardless of end goal, this would be a strong pushback on my idea. Especially since the start location is associated with a distinct cue.”]

-- Why did the authors not adopt a modeling approach to identify possible place codes in the critical task phase (https://www.ncbi.nlm.nih.gov/pubmed/21924359; https://www.ncbi.nlm.nih.gov/pmc/articles/PMC4692520/)? Given the authors' point that the hippocampus is strongly reactive to visual information and to navigation in their task, one might be quite concerned about post-arrival activity being affected by residual processing from the preceding navigation and location decision-making events. Without an explicit attempt to parse the holding-period place code it is certainly conceivable that those other hippocampal computations wash out a subtle current-position code (especially if participants do not need to process the current position following the decision - or much during navigation).

Minor concerns: I have two relatively minor concerns - I find the use of the AAL atlas on normalized brains problematic. Although this may well have been done in prior motivating studies, the AAL hippocampal mask is inaccurate (incorporating voxels from neighboring csf and cortex), and spatial normalization could further smooth and alter a voxel-wise hippocampal place code, if any. If the authors warp the AAL mask into native subject space, and touch it up (ITK-SNAP) their findings would be more anatomically precise. I also found the cross-validation approach surprising. If the authors want to ensure independence of data, why did the authors use 10-fold x-validation on a design with 5 scan sessions. Ideally, this would be conducted with a 5-fold (leave one run out) procedure, and scrambling of regressors to generate a null classification distribution occurring separately within each run (to avoid asymmetric clustering of scrambled labels on a run-wise basis - which could provide “above chance” classification).
